# Indication of Cross-Species Transmission of Astrovirus Associated with Encephalitis in Sheep and Cattle

**DOI:** 10.3201/eid2309.170168

**Published:** 2017-09

**Authors:** Céline L. Boujon, Michel C. Koch, Daniel Wüthrich, Simea Werder, Dennis Jakupovic, Rémy Bruggmann, Torsten Seuberlich

**Affiliations:** University of Bern, Bern, Switzerland

**Keywords:** meningitis/encephalitis, sheep, cattle, *Astroviridae*, neurology, ruminants, metagenomics, viruses, astrovirus, cross-species transmission, enteric infections, zoonoses

## Abstract

We report the identification of a neurotropic astrovirus associated with encephalitis in a sheep. This virus is genetically almost identical to an astrovirus recently described in neurologically diseased cattle. The similarity indicates that astroviruses of the same genotype may cause encephalitis in different species.

Astroviruses are nonenveloped viruses with a single stranded, positive-sense RNA genome. They are best known to be associated with gastroenteritis, especially in humans. Recently, reports of these viruses in association with encephalitis have increased dramatically, with reports of cases in humans (*1*), mink (*2*), and cattle (*3**–**6*).

The most common causes for viral encephalitis in sheep include maedi-visna virus, Borna disease virus, and rabies virus. In a high proportion of cases of nonsuppurative encephalitis (which is indicative of a viral infection) in sheep, however, the etiologic agent remains unknown (*7*). To investigate that matter, we subjected 3 ovine encephalitis samples from our archives to next-generation sequencing and a bioinformatics pipeline for virus discovery (Technical Appendix). In 1 animal (ID 41669), 1 of the contigs obtained had high similarity (>98%) to bovine astrovirus CH15 (BoAstV-CH15), a virus found recently in 2 cases of nonsuppurative encephalitis in cattle (*5*). The affected sheep was a 7-year-old Swiss white alpine ewe that was culled for reasons other than human consumption. No other information about the clinical history of the animal was available. Histological diagnosis consisted of severe nonsuppurative meningoencephalitis. Routine diagnostic tests for Borna, rabies, and maedi-visna viruses were all negative.

We used primers based on the BoAstV-CH15 genome sequence (GenBank accession no. KT956903) and the Sanger method to sequence the complete genome of the ovine strain (Technical Appendix Figure 1), which we named ovine astrovirus CH16 (OvAstV-CH16). The sequence we obtained shared >98% identity with BoAstV-CH15 on the nucleotide and amino acid level (Genbank accession no. KY859988; Technical Appendix Table 1). The virus reported here is genetically clearly distinct from intestinal OvAstV strains described previously (OvAstV-1 and OvAstV-2; Technical Appendix Table 1).

A phylogenetic comparison confirmed the close relationship of OvAstV-CH16 with BoAstV-CH15 (*5*) and BoAstV-BH89/14, another astrovirus detected in association with encephalitis in a cow in Germany (*6*). Recently, 2 astroviruses were reported in association with encephalitis in sheep in Scotland (OvAstV UK/2013/ewe/lib01454 and UK/2014/lamb/lib01455) (*8*), and we included their genomic data in the study comparison. All these strains clustered in the same branch of the phylogenetic tree, with >95% amino acid sequence similarity in the viral capsid protein (Technical Appendix Figure 2) and, therefore, should be considered 1 genotype species (*9*). When we compared all these viruses more closely on the amino acid level, we were not able to find any sequence variant that could be specifically associated with a tropism for sheep or cattle (Technical Appendix Table 3).

We then analyzed brain samples of sheep with nonsuppurative encephalitis of unknown etiology (n = 47), which had been identified within the framework of active disease surveillance in Switzerland (*7*), by RT-PCR specific for BoAstV-CH15 (online Technical Appendix). None reacted positively, implying a low incidence of OvAstV-CH16 infection associated with encephalitis in the sheep population in Switzerland.

To confirm the presence of OvAstV-CH16 in situ, we used polyclonal antisera targeted at the putative capsid protein of BoAstV-CH15 and tested formalin-fixed, paraffin-embedded brain tissues of sheep 41669 by immunohistochemistry (Technical Appendix). We observed positive staining of neurons, assessed as such on the basis of morphological criteria, in all brain regions examined (e.g., medulla oblongata, cerebellum, thalamus, hippocampus, cortex, caudate nucleus), in particular in some areas (Figure; Technical Appendix Figure 3). This finding supports a plausible biological association of OvAstV-CH16 infection and encephalitis in the sheep under investigation and underlines again the close relationship between OvAstV-CH16 and BoAstV-CH15. The identification of similar astroviruses in sheep and cattle with comparable diseases, by different methods and in distinct geographic areas, further strengthens these findings. We consider it unlikely that the ovine cases reported in Scotland and Switzerland are epidemiologically related and speculate that the respective viruses were already geographically widely spread but were undetected until recently, which also seems to be the case for neurotropic astroviruses in cattle.

**Figure:**
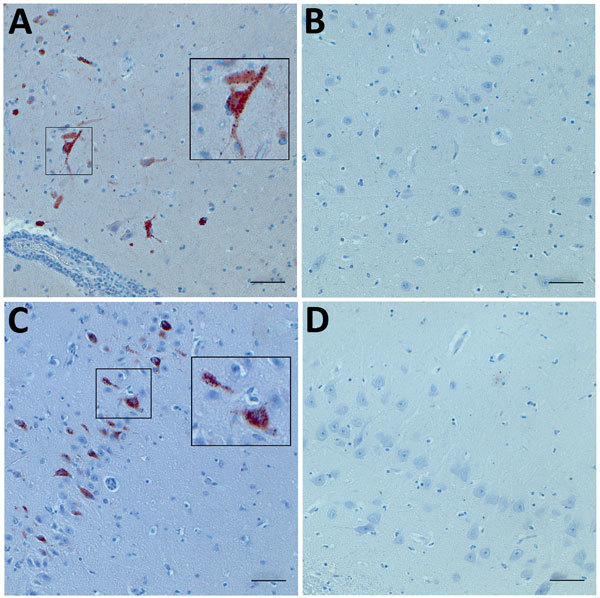
Immunohistochemistry (IHC) for ovine astrovirus CH16 in brain tissues (hippocampus) of a sheep (ID 41669) with encephalitis using 2 polyclonal antisera targeted at the putative capsid protein of bovine astrovirus CH15. A) IHC using antiserum against the conserved region of the capsid protein showing positive staining (box at left; box at right shows area at higher magnification); B) negative control. C) IHC using antiserum against the variable regions of the capsid protein n showing positive staining (box at left; box at right shows area at higher magnification); D) negative control. Scale bars indicate 50 μm.

The importance of the link between astroviruses and encephalitis is increasingly being brought to light, but the factors determining their tropism and neuroinvasion are still unknown. Deeper epidemiologic, genetic, and molecular investigations will help to clarify these aspects of astrovirus pathology. Astroviruses were traditionally considered to be host specific, but in recent years, several reports challenged this assumption; for instance, when human astroviruses were found in fecal samples of primates (*10*). In such cases, however, effective infection of atypical hosts was never shown. In this study, we demonstrated the presence of the virus in situ, a finding that strengthens the likelihood of such an infectious event. The fact that a virus of the same genotype was described in cattle with similar pathologic findings also challenges this concept of host specificity and suggests that astroviruses can cross the species barrier and, therefore, represent a zoonotic threat as not only a gastroenteritic agent but also a potential cause of encephalitis.

Technical Appendix Additional materials and details for study of astrovirus associated with encephalitis in a sheep.

## References

[1] Vu DL, Cordey S, Brito F, Kaiser L. Novel human astroviruses: Novel human diseases? J Clin Virol. 2016;82:56–63. 10.1016/j.jcv.2016.07.004 27434149

[2] Blomström AL, Widén F, Hammer AS, Belák S, Berg M. Detection of a novel astrovirus in brain tissue of mink suffering from shaking mink syndrome by use of viral metagenomics. J Clin Microbiol. 2010;48:4392–6. 10.1128/JCM.01040-10 20926705 PMC3008476

[3] Li L, Diab S, McGraw S, Barr B, Traslavina R, Higgins R, et al. Divergent astrovirus associated with neurologic disease in cattle. Emerg Infect Dis. 2013;19:1385–92. 10.3201/eid1909.130682 23965613 PMC3810933

[4] Bouzalas IG, Wüthrich D, Walland J, Drögemüller C, Zurbriggen A, Vandevelde M, et al. Neurotropic astrovirus in cattle with nonsuppurative encephalitis in Europe. J Clin Microbiol. 2014;52:3318–24. 10.1128/JCM.01195-14 24989603 PMC4313157

[5] Seuberlich T, Wüthrich D, Selimovic-Hamza S, Drögemüller C, Oevermann A, Bruggmann R, et al. Identification of a second encephalitis-associated astrovirus in cattle. Emerg Microbes Infect. 2016;5:e5. 10.1038/emi.2016.5 26785943 PMC5603821

[6] Schlottau K, Schulze C, Bilk S, Hanke D, Höper D, Beer M, et al. Detection of a novel bovine astrovirus in a cow with encephalitis. Transbound Emerg Dis. 2016;63:253–9. 10.1111/tbed.12493 26948516

[7] Oevermann A, Botteron C, Seuberlich T, Nicolier A, Friess M, Doherr MG, et al. Neuropathological survey of fallen stock: active surveillance reveals high prevalence of encephalitic listeriosis in small ruminants. Vet Microbiol. 2008;130:320–9. 10.1016/j.vetmic.2008.01.015 18355992

[8] Pfaff F, Schlottau K, Scholes S, Courtenay A, Hoffmann B, Höper D, et al. A novel astrovirus associated with encephalitis and ganglionitis in domestic sheep. Transbound Emerg Dis. 2017;64:677–82. 10.1111/tbed.12623 28224712

[9] Bosch A, Pintó RM, Guix S. Human astroviruses. Clin Microbiol Rev. 2014;27:1048–74. 10.1128/CMR.00013-14 25278582 PMC4187635

[10] Karlsson EA, Small CT, Freiden P, Feeroz MM, Matsen FA IV, San S, et al. Non-human primates harbor diverse mammalian and avian astroviruses including those associated with human infections. PLoS Pathog. 2015;11:e1005225. 10.1371/journal.ppat.1005225 26571270 PMC4646697

