# Supplementary material for: Indication of Cross-Species Transmission of Astrovirus Associated with Encephalitis in Sheep and Cattle
Source: Emerg Infect Dis. 2017 Sep;23(9):1604–6. doi: 10.3201/eid2309.170168 (PMC5572871; doi:10.3201/eid2309.170168)
Supplement: Technical Appendix — Additional materials and details for study of astrovirus associated with encephalitis in a sheep. [file 17-0168-Techapp-s1.pdf]

Article DOI: https://doi.org/10.3201/eid2309.170168

Indication of Cross-Species Transmission of Astrovirus Associated with Encephalitis in Sheep and Cattle

Technical Appendix

Tissue Samples

Sheep tissue samples were collected in the framework of a neuropathological survey in the small ruminant population in Switzerland in 2004 and 2005 (*1*). Whole brains of sheep and goats that died on farm for unknown reasons were collected according to a standardized procedure. Brains were cut sagittally into two equal halves. One half was stored at −20°C for the purpose of molecular testing. The other half was fixed in formalin, and representative brain regions were paraffin embedded and processed for histopathology. Brain sections were then HE stained and examined for histopathological lesions indicative of neurologic disease. Those samples with lesions consistent with a viral infection of the brain, i.e., a nonsuppurative inflammatory pattern, were further tested for common viral pathogens associated with encephalitis in sheep and goats (small ruminant lentiviruses, Borna virus, rabies virus). Tissues were archived in the Biobank of the Division of Neurologic Sciences, Vetsuisse Faculty, University of Bern, until further analysis in the current study.

Next-Generation Sequencing

DNA and RNA extracts from frozen brain tissues of 3 sheep diagnosed with severe nonsuppurative encephalitis, but with negative test results for the above-mentioned viruses, were processed to next-generation-sequencing libraries separately (*2*). Libraries were sequenced in an Illumina HiSeq using 1 lane of paired-end 150 bp reads, with yields of 177,146,907–195,143,918 reads for the DNA extracts and 113,160,073–251,062,995 reads for the RNA extracts. The reads of the DNA libraries were mapped to the *Ovis aries* reference genome (version 3.1) using Bowtie 2 (version 2.2.4) (*3*) and the reads of the RNA libraries were mapped to the *Ovis aries* reference

genome (version 3.1) using STAR (version 2.5.0b) (*4*). The reads that did not align to the reference genome were quality trimmed using Trimmomatic (version 0.33, option: SLIDINGWINDOW:4:14 MINLEN:127) (*5*). The trimmed reads were assembled using SPAdes (version 3.9.0, options:–meta -k 21,33,55,77,99,127) (*6*). The resulting assembly was subsequently analyzed using the virus discovery pipeline described by Wüthrich et al. (*7*).

Sanger Sequencing

The whole genome length of the virus was Sanger sequenced with primers that were designed on the sequence of BoAstV-CH15. Briefly, RNA was extracted from brain tissue of animal 41669 with TRI reagent (Sigma-Aldrich). Using primers BoAstV 15 2do, BoAstV 15 3do, BoAstV 15 q1do, BoAstV 15 q2do (Technical Appendix Table 3), and oligo(dT), viral RNA was reverse transcribed with ThermoScript Reverse Transcriptase (Life Technologies). Using different combinations of primers recognizing sequences scattered over the whole genome length of BoAstV-CH15, binding on both strands of the cDNA and spaced apart from one another with ~500 nt (the sequence of those primers are available on demand), PCR amplified fragments were directly sequenced in both directions, until the whole genome sequence of the new virus was covered.

Sequence Comparisons and Phylogenetic Analysis

The obtained full-length sequence was compared with full-length sequences of BoAstV-CH15 (*8*), BoAstV-BH89/14 (*9*), OvAstV-UK/2013/ewe/lib01454 and OvAstV-UK/2014/lamb/lib01455 (*10*), and 2 ovine astrovirus strains that were detected in feces samples of sheep (*11*, *12*) (Technical Appendix Table 1 and Technical Appendix Figure 2), using the alignment function of the Geneious software Version 9.1.5 (Biomatters). The phylogenetic tree was constructed from amino acid sequences of the astrovirus capsid proteins by maximum-likelihood using the PHYML plug-in in Geneious with 100 bootstraps.

RT-PCR Screening

Forty-seven brain homogenates of sheep diagnosed with nonsuppurative encephalitis, which were available in our archives from another study (*1*), were tested by RT-PCR with

primers BoAstV CH15 3fo and BoAstV CH15 3do (*8*) (Technical Appendix Table 2) using the OneTaq One-Step RT-PCR Kit (New England Biolabs), with the alternative protocol described by the manufacturer. Cycling conditions were as follows: 48°C for 15 min, 94°C for 1 min, 40 cycles of 94°C for 15 sec, 52°C for 30 sec and 68°C for 45 sec, and 68°C for 5 min.

Antibody Production

The methods used to obtain antibodies were the same as those we described previously for another bovine astrovirus, BoAstV-CH13 (*13*). Briefly, rabbits were immunized with recombinant viral proteins expressed in bacteria and the resulting hyperimmune serum samples were collected and affinity purified. Only diverging elements will be mentioned here. First, because structural proteins are usually produced in greater amounts than nonstructural ones during viral replication, we chose only 2 regions on the corresponding putative open reading frame (ORF), ORF2, similar to ORF2-con and ORF2-var described in the previous study. CH15-ORF2-con corresponds to the conserved region, and N-terminal part of protein Vp90, CH15-ORF2-var to a more variable, more C-terminally located part of this protein (*14,15*) (Technical Appendix Figure 1). Reverse transcription was performed with oligo(dT). The primers subsequently used to amplify the regions of interest were the following: CH15O2c NheI F and CH15O2c *Bam*HI R for the CH15-ORF2-con region and CH15O2v NheI F and CH15O2v *Bam*HI R for CH15-ORF2-var (Technical Appendix Table 3). Lysis of transformed bacteria was performed using 1 mg/l Lysozyme from chicken egg white (Sigma Aldrich), and buffers used for protein purification consisted in PBS with 1 M urea, the concentration of imidazole used in the binding/washing buffer being 10 mM and 20 mM, respectively. One rabbit was injected subcutaneously with 50–200 µg of each antigen contained in SDS-PAGE gel pieces (LowDose Antiserum protocol).

Immunohistochemistry

For both antisera, a simple screening with 3 different antigen retrieval methods (microwave heating in citrate buffer at pH 6 and pH 9, proteinase K treatment) and dilutions (1:20, 1:50, 1:100) was sufficient to determine the best performing parameters, which were microwave heating in citrate buffer at pH 6 and 1:50 dilution of the primary antibody, the

protocol being otherwise the same as the one described in another of our studies (*13*). Each method was tested on case 41669 and a negative control sheep which was negative for OvAstV-CH16 by RT-PCR.

References

1. Oevermann A, Botteron C, Seuberlich T, Nicolier A, Friess M, Doherr MG, et al. Neuropathological survey of fallen stock: active surveillance reveals high prevalence of encephalitic listeriosis in small ruminants. Vet Microbiol. 2008;130:320–9. PubMed http://dx.doi.org/10.1016/j.vetmic.2008.01.015

2. Bouzalas IG, Wüthrich D, Walland J, Drögemüller C, Zurbriggen A, Vandevelde M, et al. Neurotropic astrovirus in cattle with nonsuppurative encephalitis in Europe. J Clin Microbiol. 2014;52:3318–24. PubMed http://dx.doi.org/10.1128/JCM.01195-14

3. Langmead B, Salzberg SL. Fast gapped-read alignment with Bowtie 2. Nat Methods. 2012;9:357–9. PubMed http://dx.doi.org/10.1038/nmeth.1923

4. Dobin A, Davis CA, Schlesinger F, Drenkow J, Zaleski C, Jha S, et al. STAR: ultrafast universal RNA-seq aligner. Bioinformatics. 2013;29:15–21. PubMed http://dx.doi.org/10.1093/bioinformatics/bts635

5. Bolger AM, Lohse M, Usadel B. Trimmomatic: a flexible trimmer for Illumina sequence data. Bioinformatics. 2014;30:2114–20. PubMed http://dx.doi.org/10.1093/bioinformatics/btu170

6. Bankevich A, Nurk S, Antipov D, Gurevich AA, Dvorkin M, Kulikov AS, et al. SPAdes: a new genome assembly algorithm and its applications to single-cell sequencing. J Comput Biol. 2012;19:455–77. PubMed http://dx.doi.org/10.1089/cmb.2012.0021

7. Wüthrich D, Boujon CL, Truchet L, Selimovic-Hamza S, Oevermann A, Bouzalas IG, et al. Exploring the virome of cattle with non-suppurative encephalitis of unknown etiology by metagenomics. Virology. 2016;493:22–30. PubMed http://dx.doi.org/10.1016/j.virol.2016.03.009

8. Seuberlich T, Wüthrich D, Selimovic-Hamza S, Drögemüller C, Oevermann A, Bruggmann R, et al. Identification of a second encephalitis-associated astrovirus in cattle. Emerg Microbes Infect. 2016;5:e5. PubMed http://dx.doi.org/10.1038/emi.2016.5

9. Schlottau K, Schulze C, Bilk S, Hanke D, Höper D, Beer M, et al. Detection of a novel bovine astrovirus in a cow with encephalitis. Transbound Emerg Dis. 2016;63:253–9. PubMed http://dx.doi.org/10.1111/tbed.12493

10. Pfaff F, Schlottau K, Scholes S, Courtenay A, Hoffmann B, Höper D, et al. A novel astrovirus associated with encephalitis and ganglionitis in domestic sheep. Transbound Emerg Dis. 2017;64:677–82. PubMed http://dx.doi.org/10.1111/tbed.12623

11. Jonassen CM, Jonassen TØ, Sveen TM, Grinde B. Complete genomic sequences of astroviruses from sheep and turkey: comparison with related viruses. Virus Res. 2003;91:195–201. PubMed http://dx.doi.org/10.1016/S0168-1702(02)00269-1

12. Reuter G, Pankovics P, Delwart E, Boros Á. Identification of a novel astrovirus in domestic sheep in Hungary. Arch Virol. 2012;157:323–7. PubMed http://dx.doi.org/10.1007/s00705-011-1151-4

13. Boujon CL, Selimovic-Hamza S, Bouzalas I, Seuberlich T. Development and validation of an immunohistochemistry procedure for the detection of a neurotropic bovine astrovirus. J Virol Methods. 2017;239:26–33. PubMed http://dx.doi.org/10.1016/j.jviromet.2016.10.013

14. Wang QH, Kakizawa J, Wen LY, Shimizu M, Nishio O, Fang ZY, et al. Genetic analysis of the capsid region of astroviruses. J Med Virol. 2001;64:245–55. PubMed http://dx.doi.org/10.1002/jmv.1043

15. Bass DM, Qiu S. Proteolytic processing of the astrovirus capsid. J Virol. 2000;74:1810–4. PubMed http://dx.doi.org/10.1128/JVI.74.4.1810-1814.2000

Technical Appendix Table 1. Pairwise sequence similarities of neurotropic astrovirus strains in ruminants.

	Nucleotide / amino acid similarity, %						
ORF	BoAstV-BH89/14	BoAstV-CH15	**OvAstV-CH16***	OvAstV-1	OvAstV-2	UK/2013/ewe	UK/2014/lamb
ORF 1a							
BoAstV-BH89/14	-	98.6	**98.5**	72.7	n.a.	98.8	98.9
BoAstV-CH15	91.1	-	**98.9**	72.2	n.a.	98.6	98.5
**OvAstV-CH16***	**91.4**	**98.3**	**-**	**72.5**	**n.a.**	**98.2**	**98.3**
OvAstV-1	68.0	67.6	**67.8**	-	n.a.	72.2	72.3
OvAstV-2	n.a.	n.a.	**n.a.**	n.a	-	n.a	n.a
UK/2013/ewe	94.3	91.4	**91.5**	68.0	n.a.	-	99.9
UK/2014/lamb	94.4	91.3	**91.6**	68.0	n.a.	99.7	-
ORF1ab							
BoAstV-BH89/14	-	99.0	**98.9**	76.6	n.a.	99.0	76.6
BoAstV-CH15	92.4	-	**99.2**	76.3	n.a.	98.7	98.6
**OvAstV-CH16**	**92.3**	**98.6**	**-**	**76.6**	**n.a.**	**98.5**	**98.5**
OvAstV-1	70.0	70.4	**70.2**	-	n.a.	76.5	76.6
OvAstV-2	n.a.	n.a.	**n.a.**	n.a.	-	n.a.	n.a.
UK/2013/ewe	94.3	91.8	**91.8**	70.0	n.a.	-	99.9
UK/2014/lamb	94.3	92.1	**92.1**	70.0	n.a.	99.9	-
ORF2							
BoAstV-BH89/14	-	98.7	**99.3**	72.2	26.3	95.0	95.1
BoAstV-CH15	94.9	-	**99.0**	72.2	26.2	94.6	94.7
**OvAstV-CH16**	**94.7**	**99.3**	**-**	**72.5**	**26.2**	**95.0**	**95.1**
OvAstV-1	69.3	69.5	**69.4**	-	23.8	73.1	73.3
OvAstV-2	41.0	41.5	**41.6**	40.3	-	25.1	25.3
UK/2013/ewe	87.9	88.3	**88.3**	69.8	42.0	-	99.9
UK/2014/lamb	87.9	88.3	**88.2**	69.7	42.0	99.9	-

ORF: open reading frame. White cells: nucleotide similarity. Grey cells: amino acid similarity.
*Ovine astrovirus-CH16 (OvAstV CH16, in bold) shows high similarities with the neurotropic bovine astrovirus strains (BoAstV-BH89/14, BoAstV-CH15) and the neurotropic ovine strains (UK/2013/ewe, UK/2014/lamb), but not with the ovine feces strains (OvAstV-1, OvAstV-2).

Technical Appendix Table 2. Amino acid polymorphisms among neurotropic astrovirus strains in ruminants

nsp1ab	BoAstV-CH15	BoAstV-BH89/14	UK/2013/ewe	UK/2014/lamb	OvAstV-CH16
a.a.					
35	A	S	S	S	A
104	A	V	A	A	A
110	R	K	K	K	R
211	I	F	I	I	I
212	M	V	V	V	V
223	Q	R	Q	Q	Q
253	M	V	M	M	M
258	K	N	N	N	N
264	T	T	A	A	T
301	Y	Y	Y	Y	H
457	S	S	P	P	S
476	H	H	Y	Y	H
497	A	A	V	V	A
581	G	V	V	V	V
584	I	I	T	T	I
631	A	T	T	T	T
652	A	T	T	T	A
689	Y	Y	Y	Y	H
694	E	E	E	E	D
848	A	A	A	A	V
928	N	N	D	D	N
952	D	D	D	D	E
1081	V	V	I	I	V
1191	M	I	I	I	M
1200	N	N	N	N	S
1127	S	S	A	A	S
capsid	BoAstV-CH15	BoAstV-BH89/14	UK/2013/ewe	UK/2014/lamb	OvAstV-CH16
a.a.					
34	N	N	I	I	N
40	A	A	S	S	A
46	T	T	A	A	T
48	P	S	P	P	S
50	I	I	F	F	I
52	A	A	T	T	A
54	F	F	S	S	F
55	V	V	I	I	V
164	V	V	A	A	V
185	I	T	T	T	I
302	S	F	S	S	S
400	T	T	A	A	T
413	C	Y	Y	Y	Y
430	T	T	A	A	T
438	A	A	K	K	A
440	V	V	M	M	V
448	T	T	G	G	T
449	S	S	P	P	S
451	I	T	T	T	T
460	G	G	S	S	G
465	V	V	I	I	V
469	Y	Y	F	F	Y
516	A	A	S	S	A
518	T	T	S	S	T
524	V	V	I	I	V
531	V	V	I	I	V
554	N	N	D	N	N
555	R	R	Q	Q	R
563	E	E	D	D	E
564	T	T	P	P	T
565	S	S	A	A	S
575	M	M	I	I	M
579	Y	Y	I	I	Y
580	G	G	N	N	G
582	T	T	A	A	T
588	I	I	V	V	I
590	V	V	F	F	V
596	I	I	F	F	I
625	K	R	K	K	K

nsp1ab	BoAstV-CH15	BoAstV-BH89/14	UK/2013/ewe	UK/2014/lamb	OvAstV-CH16
635	T	T	S	S	T
664	S	N	N	N	N
732	I	I	I	I	V
754	P	S	S	S	S

nsp1ab: nonstructural precursor protein. capsid: capsid precursor protein. a.a.: amino acid position.

Technical Appendix Table 3. Primers used in this study

Primer name	Sequence (5′-3′)	Orientation	Application
BoAstV 15 2do	GTC CCT TGA CCA TTG TTG C	Reverse	Reverse transcription
BoAstV 15 3fo	GTC TTG CGC GCT GAG C	Forward	Amplification in screening RT-PCR
BoAstV 15 3do	TGG GTA ATT CTC TAA GCT GTA CTT	Reverse	Reverse transcription and amplification in screening RT-PCR
BoAstV 15 q1do	AAG CTC GAA TTC GCT CCT TCT TAA CCT TAG AGT TAA C	Reverse	Reverse transcription
BoAstV 15 q2do	AGA CTC GAA TTC GGA GGT GTA GGG TAC TGC	Reverse	Reverse transcription
CH15O2c NheI F	ATC GAG CTA GCA AGG GAG GAC CAA AGT TTG AC	Forward	Amplification of ORF2-con fragment
CH15O2c *Bam*HI R	CGA CTG GAT CCA GCC AGC GAT GCA TAT ACA AG	Reverse	Amplification of ORF2-con fragment
CH15O2v NheI F	ATC GAG CTA GCG ACT TCT CAG CCA GCC CTT G	Forward	Amplification of ORF2-var fragment
CH15O2v *Bam*HI R	CGA CTG GAT CCT TCT GAG GAC GAC CCA GAC	Reverse	Amplification of ORF2-var fragment

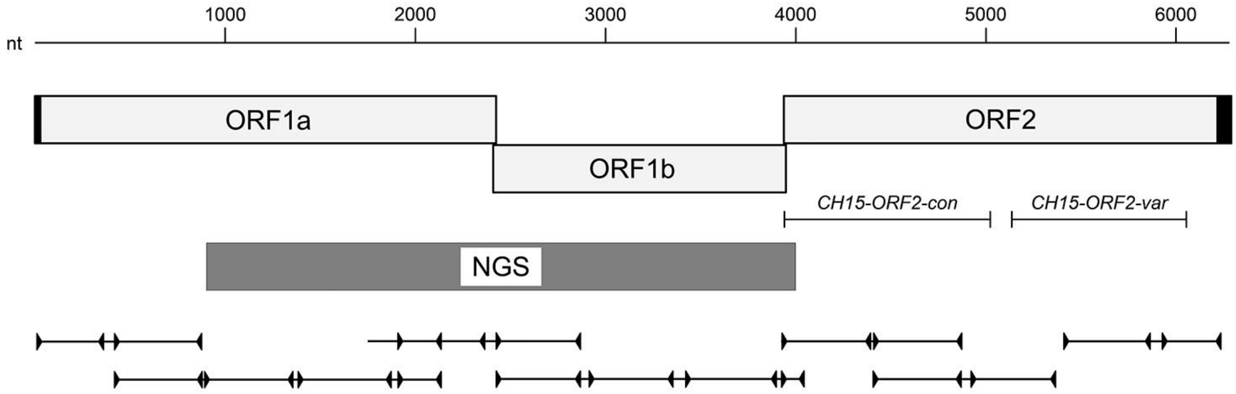

Technical Appendix Figure 1. Sequencing information obtained by next-generation sequencing (NGS) investigation of the affected sheep (ID 41669), related to the genomic structure of bovine astrovirus CH15. nt: nucleotide position. ORF: open reading frame. Black rectangles: untranslated regions. Dark gray rectangle: contiguous sequence (contig) obtained by NGS. Black lines delimited by bars: genome regions used for the expression of recombinant antigens in antibodies production. Black lines with arrow heads: cDNA fragments obtained by reverse transcription and used for Sanger sequencing. Arrow heads: primer binding sites and direction of Sanger sequencing.

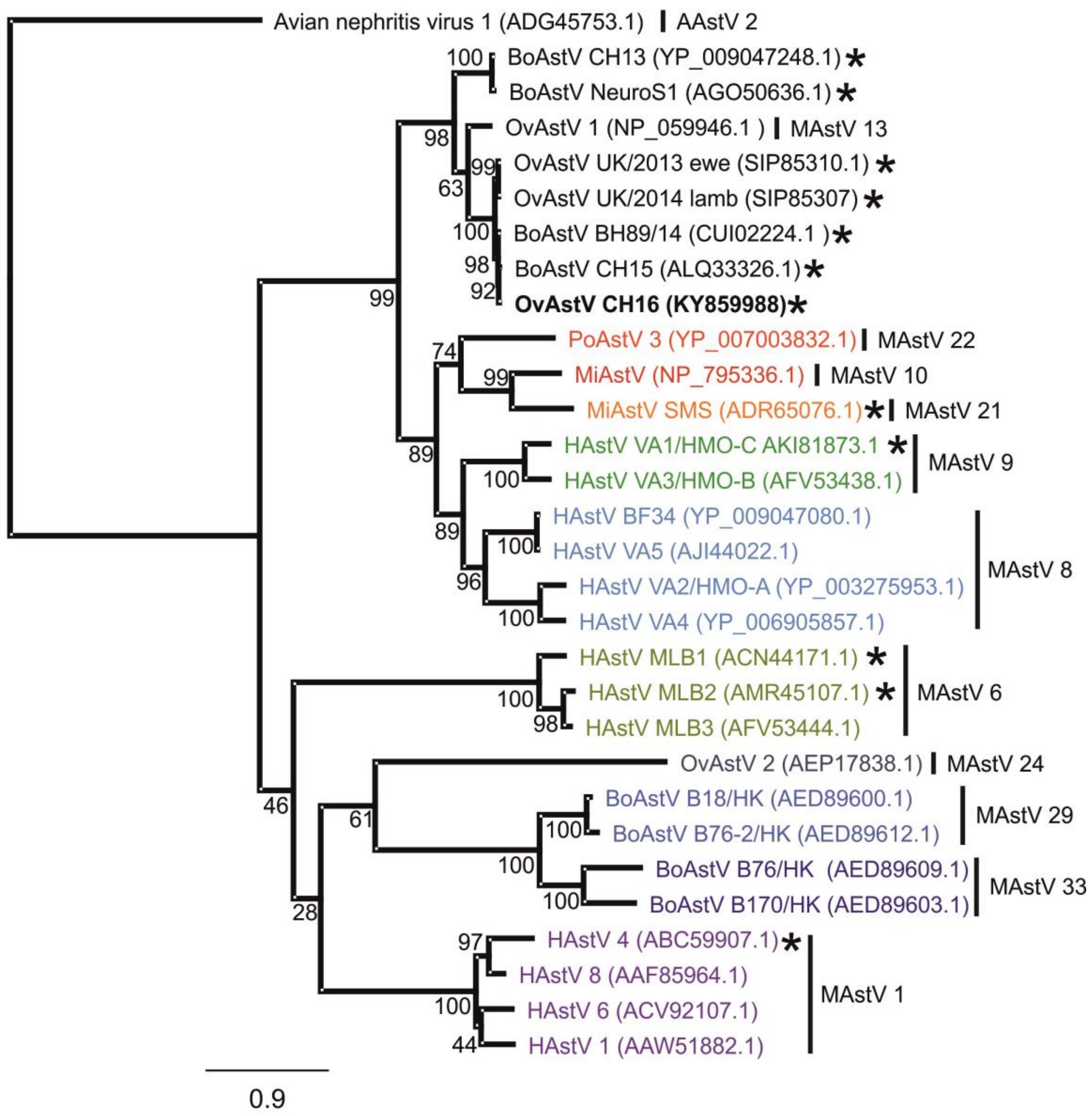

Technical Appendix Figure 2. Phylogenetic comparison of the putative capsid protein sequence of representative *Astroviridae* genotype species. The virus found in association with encephalitis in a sheep (OvAstV-CH16) is depicted in bold. Colors indicate viruses that belong to the same astrovirus species. Asterisks signal astrovirus strains that were found in association with encephalitis. HAstV: human astrovirus; PoAstV: porcine astrovirus; MiAstV: mink astrovirus; BoAstV: bovine astrovirus; MAstV: mamastrovirus; AAstV: avastrovirus.

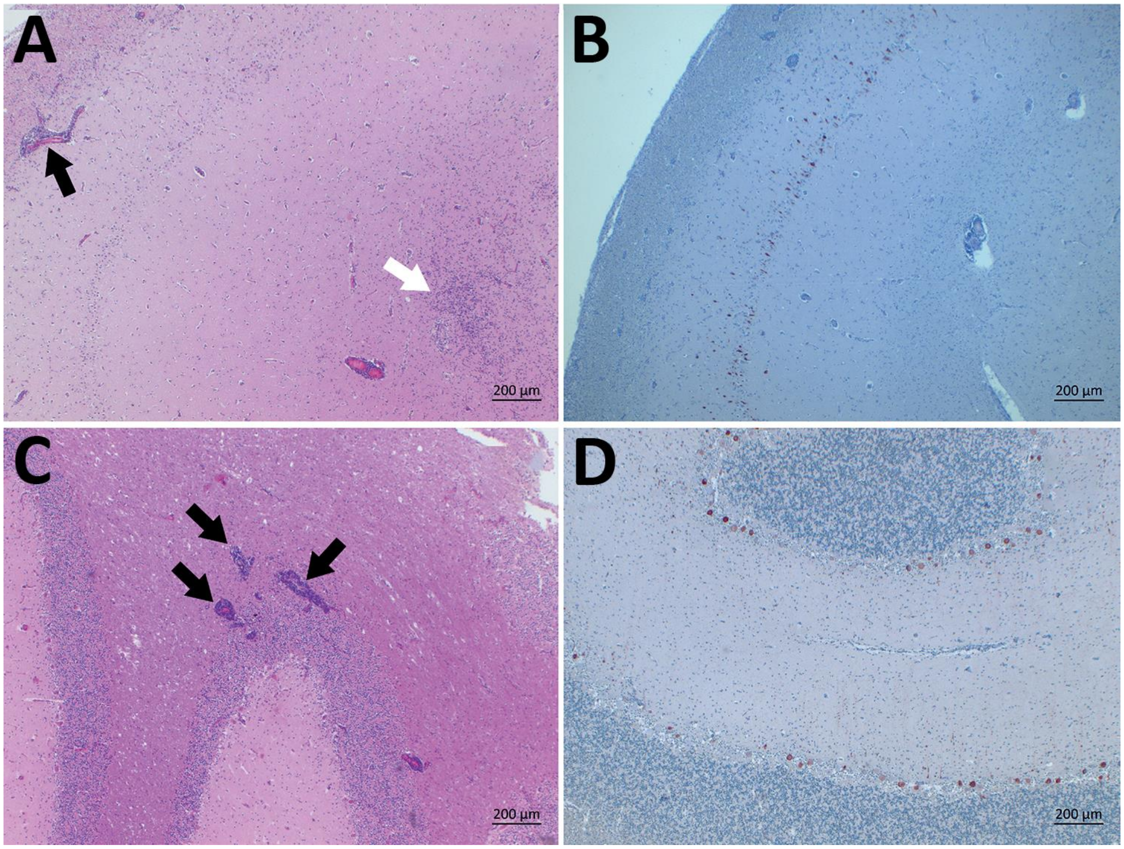

Technical Appendix Figure 3. Correlation of histological lesions with immunohistochemistry (IHC) findings in the sheep positive for ovine astrovirus CH16 (ID 41669). A. Hippocampus, hematoxylin-eosin staining. The animal suffered from severe nonsuppurative encephalitis: marked perivascular cuffing (black arrow) and gliosis (white arrow) are characteristic lesions that can be seen here. B. Hippocampus, IHC with the antibodies against the variable region of the putative capsid protein of bovine astrovirus CH15 (BoAstV-CH15). Positive staining is recognizable by dark red coloring. C. Cerebellum, hematoxylin-eosin staining. Black arrows: perivascular cuffing. D. Cerebellum, IHC with the antibodies against the variable region of the putative capsid protein of BoAstV-CH15. Remarkable positive staining of Purkinje cells.
